# Is cone-beam computed tomography (CBCT) an alternative to plain radiography in assessments of dental disease? A study of method agreement in a medically compromised patient population

**DOI:** 10.1007/s00784-024-05527-3

**Published:** 2024-01-30

**Authors:** Ninita Lindfors, Annika Ekestubbe, Fredrik Frisk, Henrik Lund

**Affiliations:** 1 Institute for Postgraduate Dental Education, Jönköping, Sweden; 2 Department of Oral and Maxillofacial Radiology, Institute of Odontology, Sahlgrenska Academy, University of Gothenburg, P.O. Box 450, 405 30 Göteborg, Sweden; 3 Department of Endodontology, Institute of Odontology, Sahlgrenska Academy, University of Gothenburg, Gothenburg, Sweden; 4 School of Health and Welfare, Jönköping University, Jönköping, Sweden

**Keywords:** Cone-beam computed tomography, Panoramic radiography, Dental radiography, Dental diseases

## Abstract

**Objectives:**

Poor oral health and dental infections can jeopardize medical treatment and be life-threatening. Due to this, patients with head and neck malignancies, generalized tumor spread, organ transplant, or severe infection are referred for a clinical oral and radiographic examination. The aim of this study was to compare the diagnostic agreement of three radiographic modalities: intraoral radiographs (IO), panoramic radiographs (PX), and cone beam computed tomography (CBCT) for diagnosis of dental diseases.

**Materials and methods:**

Three hundred patients were examined with IO, PX, and CBCT. Periapical lesions, marginal bone level, and caries lesions were diagnosed separately by four oral radiologists. All observers also assessed six teeth in 30 randomly selected patients at two different occasions. Kappa values and percent agreement were calculated.

**Results:**

The highest Kappa value and percent agreement were for diagnosing periapical lesions (0.76, 97.7%), and for the assessment of marginal bone level, it varied between 0.58 and 0.60 (87.8–89.3%). In CBCT, only 44.4% of all teeth were assessable for caries (Kappa 0.68, 93.4%). The intra-observer agreement, for all modalities and diagnoses, showed Kappa values between 0.5 and 0.93 and inter-observer agreement varied from 0.51 to 0.87.

**Conclusions:**

CBCT was an alternative to IO in diagnosing periapical lesions. Both modalities found the same healthy teeth in 93.8%. All modalities were performed equally regarding marginal bone level. In caries diagnosis, artifacts were the major cause of fallout for CBCT.

**Clinical relevance:**

Intraoral radiography is the first-hand choice for diagnosing dental disease. For some rare cases where intraoral imaging is not possible, a dedicated panoramic image and/or CBCT examination is an alternative.

## Introduction

Poor oral health and dental infections can be life-threatening and jeopardize medical treatment [1–6]. Untreated dental disease may after certain medical procedures aggravate and generate bothersome sequel after treatment [7]. For example, radiotherapy or medication can lead to osteonecrosis in the jaws spontaneously, after tooth extraction or other dental surgical procedures. This condition can be painful with intraorally exposed bone lesions that are hard to treat and if untreated will progress. Radiation therapy can also induce soft tissue fibrosis that can reduce the ability to open the mouth sometimes leading to discomfort while eating, complicate obtaining good oral hygiene, or comply with dental procedures. Another sequel can be salivary gland hypofunction that can affect eating comfort and an increased risk for caries development.

Patients who are about to undergo treatment regarding head and neck malignancies, generalized tumor spread, organ transplant, or severe infection are generally more thoroughly examined both clinically and radiographically to diagnose oral disease. In Sweden, for example, health programs have been designed to make, e.g., cancer treatment nationally standardized, i.e., equal and efficient [8]. Therefore, these patients may be excluded from a more individualized approach as regards the extent of the radiographic examination, i.e., the principles of ALARA (As Low As Reasonably Achievable) or ALADAIP (As Low As Diagnostically Acceptable being Indication-oriented and Patient-specific).

Due to their medical condition, these patients are more vulnerable than a healthy population. They may suffer from generalized fatigue due to reduced lung capacity, medication, and stressed of their situation. Some of the patients with intraoral tumors suffer from intraoral pain, and others may have difficulties to open the mouth. All these conditions may influence their capability to cooperate to an intraoral radiographic examination (IO), thus impairing the image quality and the possibility to perform a correct diagnosis.

A dental radiographic examination is a crucial complement to the clinical examination to diagnose dental diseases, monitor illness over time, and choose the most appropriate treatment available and its effect in a long-term perspective. Still, a radiographic examination using intraorally placed detectors is the recommended radiographic technique in diagnosing the most common dental diseases. It may however require at least 18–20 images to fully cover the dentate areas and adjacent bone in an individual with a complete dentition (32 teeth). This procedure is time-consuming and not always pleasant for the patient. Sometimes a panoramic radiograph (PX) is needed to complement the IO for example when the intraoral technique is not feasible due to anatomical variants, reduced capacity to open the mouth, or pain secondary to intraoral tumors [9]. When correctly performed, PX provides valuable information.

Since its introduction in the late 1990s, cone-beam computed tomography (CBCT) has gradually changed the concept of dental radiographic imaging with its availability and excellent tomographic images of the dentomaxillofacial region at a relatively low radiation dose compared to medical computed tomography (CT). Today, a large number of CBCT devices from different manufacturers are available on the market, and in recent years, a new generation of these has been released. These new CBCT devices perform, besides CBCT acquisition, also panoramic and cephalometric imaging. This expanded range of applications has made them more accessible in general dentistry and may be an alternative radiographic method for patients who cannot tolerate intraorally placed detectors.

The aim of this study was therefore to compare the diagnostic agreement of three radiographic modalities: IO, PX, and CBCT for diagnosis of dental disease in medically compromised patients.

## Materials and methods

This study was approved by the regional ethical committee in Linköping, Sweden, and by the local radiation committee at Ryhov Hospital, Jönköping, Sweden, dnr 2013/256–31. The study was carried out according to guidelines and regulations of the 1964 Declaration of Helsinki.

### Patient population

To find a difference of 10% between the radiographic techniques with a significance level of 5% and a power of 80, we calculated that 300 patients had to be included in the study.

The patients were recruited in between September 2015 and November 2016, in the county of Jönköping, Sweden. All consecutive medically compromised patients who were referred for a dental radiographic examination were invited to participate in the study. To be included, the patient had to be dentated or rehabilitated with dental implants, be able to sit in a chair without support of a high neck rest, comprehend the patient information either by themselves or by an interpreter, and accept to participate.

Due to different medical diagnoses (Table 1), the patients were primarily referred from Ryhov Hospital, Region Jönköping County, Sweden, to the Department of Maxillofacial Surgery or Oral Medicine for clinical examination.

**Table 1:** Patient characteristics by age, gender, number of teeth, and dental implants, in total and by medical diagnosis

	Total	Medical diagnosis					
		Malignant disease*	Head and neck malignancy	Metastatic malignancy**	Organ transplant	Heart valve disease	Infectious disease***
Patients (*n*)	300	35	43	49	35	109	29
Age mean (min − max)	65 (18 − 93)	63 (25 − 90)	66 (23 − 93)	66 (32 − 90)	48 (18 − 71)	70 (32 − 91)	66 (29 − 89)
Female (*n*/%)	124/41	20	12	33	11	41	7
Male (*n*/%)	176/59	15	31	16	24	68	22
*Teeth*							
(*n*)	7022	909	1013	1166	904	2359	671
Mean	23.4	26.0	23.6	28.8	25.8	20.0	23.1
(Min − max)	(0 − 32)	(10 − 32)	(0 − 32)	(4 − 31)	(5 − 32)	(0 − 32)	(7 − 32)
*Dental implants*							
(*n*)	112	2	28	7	3	70	2
Mean	5.1	2.0	9.3	2.3	3.0	5.4	2.0
(Min − max)	(1 − 15)	(2 − 3)	(3 − 15)	(1 − 3)	(3 − 3)	(2 − 12)	(2 − 2)

*Different types of leukemia or myeloma

**Metastatic bone lesions. To be treated with bisphosphonate or denosumab drugs

***Endocarditis, brain abscess

All patients were then referred to the Department of Maxillofacial Radiology to undergo radiographic examination. All patients were examined with IO and PX radiographs, according to the local standard protocol for this patient population. All patients who fulfilled the criteria for inclusion were then thoroughly informed of the study: the purpose, the increased radiation dose, how data would be stored, and results presented. To those patients who accepted participation by a signed confirmation, an additional CBCT scan was performed.

### Radiographic examinations and evaluation

The IO examinations were performed applying a parallel technique using a Focus, Instrumentarium (GE Healthcare Finland) radiographic equipment together with Sirona Schick 33 sensors (Sirona Dental, Salzburg, Austria). Exposure parameters used were 60 kV, 7 mA, and exposure time varying between 0.16 and 0.25 s depending on dental region and patient size. The PX were obtained with an Orthophos XG 5 (Sirona Dental Systems, Bernsheim, Germany). Scan time was 14.1 s and exposure settings varied between 8–15 mA and 62–73 kV depending on patient size. The CBCT examinations were performed using Veraviewepocs® 3D R 100 (J. Morita Mfg. Corp. Kyoto, Japan) with a field-of-view (FOV) of 100 mm × 80 mm enclosing the complete dentition. The exposure settings were 85 kV and 5 mA and the scan time of 9.4 s was optimized for the diagnostic yield. The voxel size was 0.160 mm. Quality assessment of radiographic examinations was continuously performed by an oral radiologist according to clinical procedures for the different imaging modalities, i.e., image area, projection geometry as regards intraoral imaging and panoramic radiographs, as well as FOV, and eventual presence of motion artifacts in CBCT-examinations. Retakes were made when necessary.

The observers were four senior board-certified specialists in Dentomaxillofacial Radiology, from the Department of Oral and Maxillofacial Radiology, Institute of Odontology at the University of Gothenburg, Sweden, and from the Department of Dentomaxillofacial Radiology at the Institute for Postgraduate Dental Education in Jönköping, Sweden.

The patients were divided among the observers as follows: observer 1 was allotted the first 60 consecutive patients, observer 2 the next consecutive 60 patients, and so on. Observer 4 (principal investigator) evaluated the 120 remaining consecutive patients.

To make the radiographs (IO and PX) available for the observers in Gothenburg, images were exported in DICOM-format (Digital Imaging and Communications In Medicine) to the local PACS system (Picture Archiving and Communication System), Sectra IDS7 (Sectra Imtec AB, Linköping, Sweden). The images were displayed on two 21.3-inch color LCD monitors (EIZO RadiForce RX240, Eizo Nanao Corp., Japan) with a resolution of 1600 × 1200 pixels. In Jönköping, the radiographs were displayed on two 21-inch color LCD monitors, (MDCC-2121 Barco, Kortrijk, Belgium) with a graphic card MXRT5200 and a resolution of 1600 × 1200 pixels, using PACS (IMPAX 6.5.3 AGFA Healthcare, Belgium). The CBCT examinations were viewed using software i-Dixel-3DX (3D, version 1.691; J. Morita) and the observers were able to use the software program to align the image planes to obtain the best visualization for each diagnostic task and tooth/root. Further, adjustment of brightness and contrast was also possible.

No clinical data was available for the observers. Initially and prior to the evaluation, the observers were calibrated. The calibration was performed by joint evaluation of ten patients regarding all diagnoses to achieve consensus. Non-erupted teeth were registered as missing and all tooth surfaces had to be clearly depicted otherwise the tooth was registered as not possible to evaluate due to artifact, i.e., incorrect projection geometry, high-contrast tissues and metallic restorations, or not depicted.

For each observer, the images on all the allotted patients were evaluated separately, i.e., first the intraoral radiographs on all patients, then the panoramic, and finally the CBCT images on all patients. The assessment of the different imaging modalities was separated in time by at least 1 month.

To calculate inter- and intra-observer agreement, all observers assessed six teeth from different regions in 30 randomly selected patients from the whole patient population at two different occasions separated in time with approximately 1 month. The randomization was made using Microsoft Excel 2010 (Microsoft, Redmond, Wash, USA) and the number of teeth and patients was based on a power calculation.

To facilitate the recording of findings, a template was made using Microsoft Access Office 2010 (Microsoft Redmond, Wash, USA). Each observer had three different Access files, one for the basic number of patients (60 or 120 individuals) and two additional for calculating inter- and intra-observer agreement. Each Access file consisted of templates with all 32 teeth positions available with a corresponding square below for scoring. In every Access file, there were nine different templates, one for each modality and for the three different diagnostic tasks (periapical radiolucency, marginal bone level and caries lesions). For each patient, an overall assessment of the image quality for each radiographic method and all diagnoses was made. In total, 63,310 (63,198 teeth + 112 implants) scores were set in the main study and 432 to calculate inter- and intra-observer agreement.

### Evaluation criteria

#### Periapical lesions

The unit was the tooth regardless of the number of roots. Impacted teeth were excluded and scored missing: 1, no disease (including widened periodontal ligament); 2, disease (periapical lesion irrespective of size and/or location); 3, not possible to evaluate due to artifact; 4, not possible to evaluate due to not depicted; 5, missing tooth.

#### Marginal bone level (Tooth)

The unit was the tooth: 1, no disease (marginal bone level ≤ 5 mm from the cemento-enamel junction (CEJ); 2, disease (marginal bone level > 5 mm from CEJ); 3–5 see periapical evaluation.

#### Marginal bone level (Dental implant)

The unit was the implant: 6, no disease (marginal bone level ≤ 3 mm apical to the reference point; 7, disease (marginal bone level > 3 mm apical to the reference point); 8, not possible to evaluate due to artifact; 9, not possible to evaluate due to not depicted.

#### Caries lesions

The unit was the tooth: 1, no disease (no caries lesion including the enamel); 2, disease (caries in the dentin and/or root surface and secondary caries); 3–5 see periapical evaluation.

#### Overall score image quality

For each patient and modality, regardless of diagnostic task, all observers evaluated the image quality according to a score, excellent (1), good (2), acceptable (3), and poor (4).

### Statistics

To compare the diagnostic outcome, the Kappa value and observer agreement were calculated using SAS 9.4 TS Level 1M5 by SAS Institute Inc., Cary, NC, USA.

## Results

In Tables 2 and 3, every box represents two modalities being compared. Table 2 shows that IO had the highest score of teeth available for diagnosis in all diagnoses.

**Table 2:**
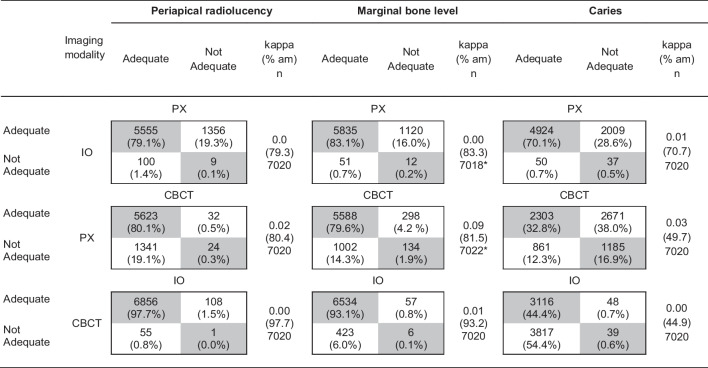
The number and percentage of teeth valid or not valid for diagnosis. Scores 1 and 2 are pooled as “adequate,” and scores 3 and 4, as “not adequate” for each diagnosis and radiographic modality (*IO* intraoral radiographs, *PX* panoramic radiograph, *CBCT* cone-beam computed tomography)

*Kappa*, kappa value; *% am*, percent agreement; *n*, number of assessments

Two teeth have been misplaced in the wrong group

**Table 3:**
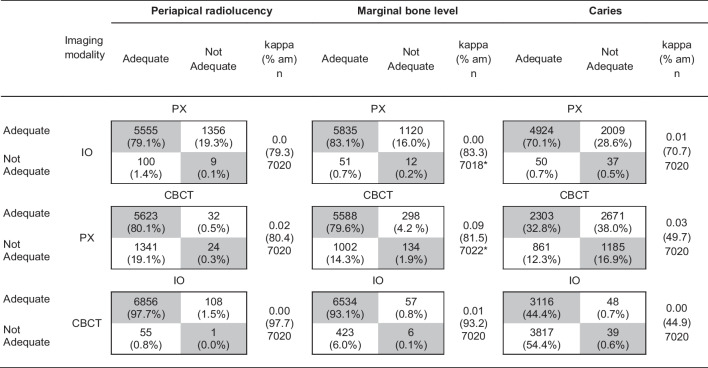
The number of scores for presence of disease (no disease score 1 and disease score 2) for each diagnosis and radiographic modality (*IO* intraoral radiographs, *PX* panoramic radiograph, *CBCT* cone-beam computed tomography)

*Kappa*, kappa value; *% am*, percent agreement; *n*, number of assessments

Table 3 consists of scores 1 and 2, “no disease” and “disease,” respectively, for the different diagnoses and modalities. Score 1 or 2 must be present in both modalities that are being compared. For example, if one tooth is scored with no disease in the IO modality, diagnosing caries, but as “not possible to evaluate due to artifact” (score 3) in the CBCT modality, the tooth is not included in the analysis.

The highest Kappa value was found diagnosing periapical radiolucency, comparing IO and CBCT (0.76). This sample group was also the largest with 6856 assessments which means that 97.7% of all 7020 teeth were assessable for diagnosis in this group. Diagnosing marginal bone level, the Kappa value varied between 0.58 and 0.60 comparing the different modalities. This group consisted of 6534 assessable teeth (93.1%). When assessing marginal bone level at dental implants, the Kappa values when comparing CBCT and PX and IO and CBCT were low, 0.18 and 0.29, respectively, representing “none to slight agreement,” and 0.43 comparing PX to IO representing “moderate agreement.” In diagnosing caries, only 44.4% of all teeth were assessable in CBCT when compared to IO (Kappa value 0.68). The Kappa value for PX and IO and CBCT and PX in diagnosing caries was 0.54 and 0.57, respectively.

**Fig. 1:**
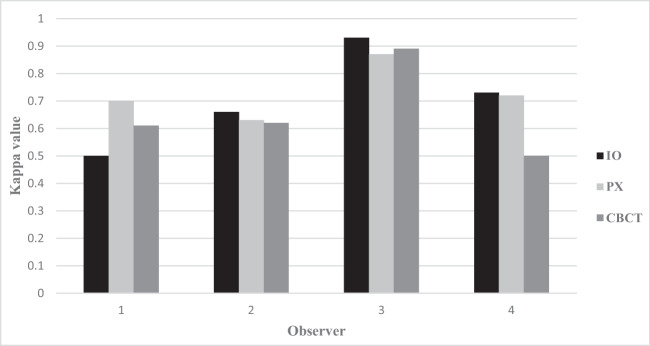
Distribution of Kappa value per observer (*n* = 4) and modality (IO, intraoral radiography; PX, panoramic; CBCT, cone-beam computed tomography) for all diagnoses (periapical radiolucency, marginal bone level, caries)

**Fig. 2:**
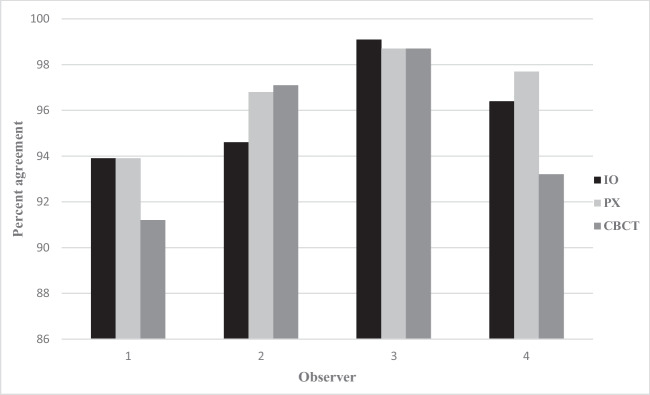
Distribution of percent agreement per observer (*n* = 4) and modality (IO, intraoral radiography; PX, panoramic; CBCT, cone-beam computed tomography) for all diagnoses (periapical radiolucency, marginal bone level, caries)

The intra-observer agreement (Figs. 1 and 2) is based on “adequate” scores, i.e., assessments of “disease” and “no disease.”

The intra-observer agreement, for all modalities and diagnoses, was for one of the observers an “almost perfect agreement” (Kappa values between 0.87 and 0.93). For the other observers, the agreement was considered “moderate” or “substantial” (Kappa values between 0.5 and 0.73).

Overall inter-observer agreement (Table 4) for each modality and diagnosis showed a variety from “moderate” to “almost perfect agreement” (Kappa value between 0.51 and 0.87).

**Table 4:** Overall inter-observer kappa and percent agreement by modality and diagnosis

	Periapical radiolucency	Marginal bone level	Caries	Periapical radiolucency	Marginal bone level	Caries
	Kappa value			Percent agreement		
IO	0.81	0.74	0.57	99.2	93.6	95.2
PX	0.51	0.80	0.53	98.1	95.6	95.6
CBCT	0.87	0.58	0.56	99.2	89.9	97.3

**Fig. 3:**
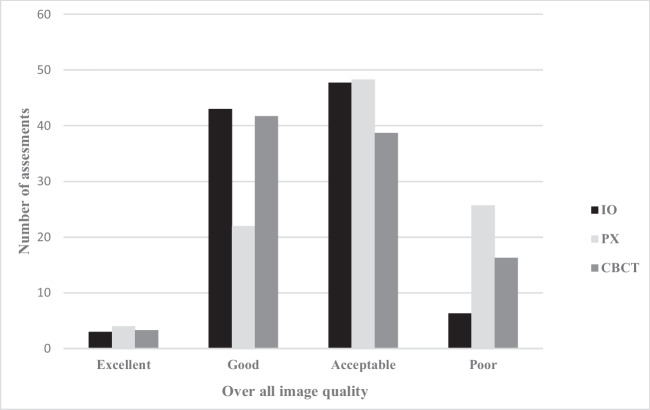
Distribution of scores of overall image quality by modality (I, intraoral radiography; PX, panoramic; CBCT, cone-beam computed tomography)

Figure 3 displays the overall image quality score. Good and acceptable image quality was the most common scores.

## Discussion

In this group of medically compromised patients, the image quality of IO was considered poor in only 6%. IO performed equally well as CBCT in diagnosing periapical diseases and with a higher agreement compared to PX and CBCT in diagnosing caries and marginal bone level. A total of 98.5% of all teeth were assessable in IO diagnosing periapical radiolucency. The intraoral technique is applicable for most patients regardless of physical condition.

In this study CBCT showed a high number of adequate scores regarding periapical but not for caries diagnosis. There are several in vitro studies comparing the diagnostic agreement of CBCT and intra-oral radiography in diagnosing caries lesions with various outcome. Osman et al. 2019 presented similar results in vitro when comparing CBCT and intra-oral radiographs influenced by different orthodontic materials causing artifacts and its effect on visualization of caries lesions. Och vilka var resultaten? Teeth lacking.

For periapical diagnoses, multiple studies report CBCT to be superior to intraoral images [10–13]. However, these studies are most often based on endodontically compromised cases, and in such cases, CBCT imaging has an impact on therapeutic decision efficacy when used in accordance with the recommendations from the European Commission guidelines. The present study compares different systems and their ability to distinguish between normal periapical status in a general population and not selected endodontic cases, and after the exclusion of images with artifacts, all systems detected an equal percentage of teeth with periapical lesions.

For diagnosis of periodontal disease, intraoral and panoramic radiographs are considered the first-hand choice. In some cases, CBCT may give additional information, e.g., for the assessment of furcation involvement and angular defects [14–16]. Nevertheless, Kim and Bassir 2017 [17] in a review article found no evidence-based guidelines in the literature supporting the necessity for CBCT imaging in periodontal treatment planning and limited evidence for diagnosis of furcation and bony defects.

In the present study, assessing marginal bone level at dental implants, the agreement between the different imaging modalities was low. This may in part be explained by the inability to identify the same dental position by the different radiographic techniques and therefore few comparisons were made (range 52–94 implants, 46–83%). However, assessing implant marginal bone level, Raes et al. 2013 [18] found that the observers underestimated the marginal bone level in CBCT images compared to intraoral images. This may reflect the examiners’ difficulty to identify the contact between bone and implant, a well-known blooming artifact. CBCT also depicts the buccal and lingual surfaces, and whenever the bone plates are very thin, they may be hard to identify as a consequence of the partial volume averaging effect. This might be the reason why in the present study, the level of agreement was minimal (Kappa 0.29) when comparing CBCT and IO.

In diagnosing caries lesions and after the exclusion of images with artifacts, all systems detected an almost equal percentage of teeth with caries lesions. However, using CBCT technique, only 45% of all teeth were assessable in comparison to IO and PX, 1.2% and 19.2%, respectively. This is mainly due to extensive artifacts from surrounding restorations and the differences in density of surrounding tissues. The observer’s judgment may also have been influenced by the inherent differences between the techniques, e.g., a summation image versus a mathematically constructed image.

In vitro studies come with a gold standard and results are presented as sensitivity, specificity, and AZ-values. This study lacks a gold standard, an inherent characteristic of the study type. However, the current study aims to verify whether there is an agreement between imaging modalities rather than determining which is superior to the other.

The rationale for using tooth as a unit for all diagnoses was to avoid bias for any of the imaging modalities. For example, in diagnosing approximal caries lesions, tooth surfaces should not overlap. This is possible to achieve with intraoral technique where the projection geometry could be individualized and more difficult using for example the panoramic technique.

In clinical practice, the detectability of, e.g., periapical lesions in intraoral radiography could be improved if the same patient has already undergone a CBCT examination. Similarly, the detectability of caries lesions in CBCT is improved if the patient has already undergone an intraoral radiographic examination. However, the aim of this study was to investigate whether a method could completely replace another, and therefore, the different imaging modalities were evaluated separately.

Values for both Kappa and percent agreement are presented due to the inequality in the different groups regarding the number of assessments. This variance can affect the outcome of the Kappa value. If observers prefer one score before the other (symmetric imbalance), the output can be a high percent agreement together with an unreasonably low Kappa. The other alternative is when the observers prefer the opposite score then the outcome can be a low percent agreement and low Kappa. If the imbalance is moderate, this may render a higher Kappa value.

One of the observers evaluated twice as many images as the others which may introduce a source of bias to the outcome. However, in this study, the number of patients included and the number of observers entail that this does not constitute any disproportionate weight in favor of any observer.

In this study, the effective dose for a CBCT examination was 0.127 mSv [19], and the panoramic images varied between 0.035 and 0.070 mSv. These dose levels were calculated using the product of the air kerma and the area of the radiation field (PKA) which was 70 mGycm^2^ at 8 mAs and 146 mGycm^2^ at 15 mAs [20] and a conversion coefficient of 500 mSv/mGycm^2^ [21]. The effective dose for a full mouth intraoral examination as conducted in the present study is approximately 0.04 mSv (with an effective dose of 0.002 mSv for one intraoral image) [22, 23]. Therefore, the choice between conventional radiography and CBCT must be individualized to be justified. Apart from a higher radiation dose, CBCT examinations are also afflicted with, for example, incremental costs for personnel, equipment, and maintenance.

Even though medically compromised, the patients in this study had an overall good dental health. Because of the risk for complications or sequel due to oral infections, these patients undergo extensive radiographic examinations with both panoramic and intraoral radiographs and sometimes even with additional CBCT according to standard protocols. Thus, this group of patients is exempted from an individual assessment of justification and optimization. The overall aim of using standard protocols for this group of patients is to ensure that the treatment is as equal and efficient as possible. On the other hand, the ALARA and ALADAIP principles are then not applied. It is therefore important to make an individual judgment for each patient on what radiographic method is efficient and justified. In this study, IO were the most efficient for all diagnoses. Whenever the patient, for various reasons, cannot tolerate IO, this study showed that CBCT could be an alternative in diagnosing periapical disease and for diagnosing marginal bone level and caries lesions if no artifacts are present.

## Conclusion

Intraoral radiography is the first-hand choice for diagnosing dental disease. For some rare cases where intraoral imaging is not possible, a dedicated panoramic image and/or CBCT examination is an alternative.

## Data Availability

No datasets were generated or analysed during the current study.
